# Surgical treatment of hepatic xanthogranuloma arising from a giant hepatic cyst causing gastrointestinal obstruction: a case report

**DOI:** 10.1093/jscr/rjac252

**Published:** 2022-07-30

**Authors:** Akihiro Kohata, Masashi Inoue, Ken Nomimura, Kazuki Matsubara, Masatoshi Kochi, Ryuichi Hotta, Kazuaki Miyamoto, Kazuhiro Toyota, Seiji Sadamoto, Tadateru Takahashi

**Affiliations:** Department of Surgery, National Hospital Organization Higashi Hiroshima Medical Center, Higashihiroshima, Japan; Department of Surgery, National Hospital Organization Higashi Hiroshima Medical Center, Higashihiroshima, Japan; Department of Surgery, National Hospital Organization Higashi Hiroshima Medical Center, Higashihiroshima, Japan; Department of Surgery, National Hospital Organization Higashi Hiroshima Medical Center, Higashihiroshima, Japan; Department of Surgery, National Hospital Organization Higashi Hiroshima Medical Center, Higashihiroshima, Japan; Department of Surgery, National Hospital Organization Higashi Hiroshima Medical Center, Higashihiroshima, Japan; Department of Surgery, National Hospital Organization Higashi Hiroshima Medical Center, Higashihiroshima, Japan; Department of Surgery, National Hospital Organization Higashi Hiroshima Medical Center, Higashihiroshima, Japan; Department of Surgery, National Hospital Organization Higashi Hiroshima Medical Center, Higashihiroshima, Japan; Department of Surgery, National Hospital Organization Higashi Hiroshima Medical Center, Higashihiroshima, Japan

**Keywords:** hepatic cyst, hepatic xanthogranuloma, gastrointestinal obstruction, laparoscopic deroofing

## Abstract

Hepatic cysts are benign liver lesions and are often asymptomatic. Large hepatic cysts may cause jaundice and portal hypertension; however, they rarely cause gastrointestinal obstruction. Symptomatic cysts require treatment, and when malignancy is suspected, cyst puncture for pathological examination of the fluid may pose a risk of dissemination. Herein, we describe a case of xanthogranuloma arising from a large hepatic cyst that was causing duodenal obstruction. Thus, cyst puncture was performed for emergency decompression. Cytological examination of the puncture fluid revealed no malignant findings. Hence, laparoscopic deroofing was performed to treat the hepatic cyst. As the cyst and duodenal wall were firmly adherent, the cyst wall was left behind without dissection from the duodenum. A two-stage approach of cyst puncture followed by surgery may be an option for patients requiring urgent treatment for potentially malignant hepatic cysts.

## INTRODUCTION

Hepatic cysts are mostly benign, asymptomatic, incidentally diagnosed, and do not require intervention. Treatment is indicated when they become symptomatic, cause complications or demonstrate rapid growth [1, 2]. Several treatment options are available, including simple aspiration, sclerotherapy and fenestration [1]. Recently, laparoscopic deroofing is increasingly used because it is minimally invasive and has a low recurrence rate [3]. The standard treatment for potentially malignant liver cysts is hepatic resection [4]. When a large hepatic cyst with malignant potential adversely affects the patient’s condition, urgent hepatectomy may be too invasive. Herein, we report a case in which puncture aspiration was performed as an urgent treatment for a large hepatic cyst causing duodenal obstruction, and laparoscopic deroofing was performed as curative therapy.

## CASE REPORT

**Figure 1:**
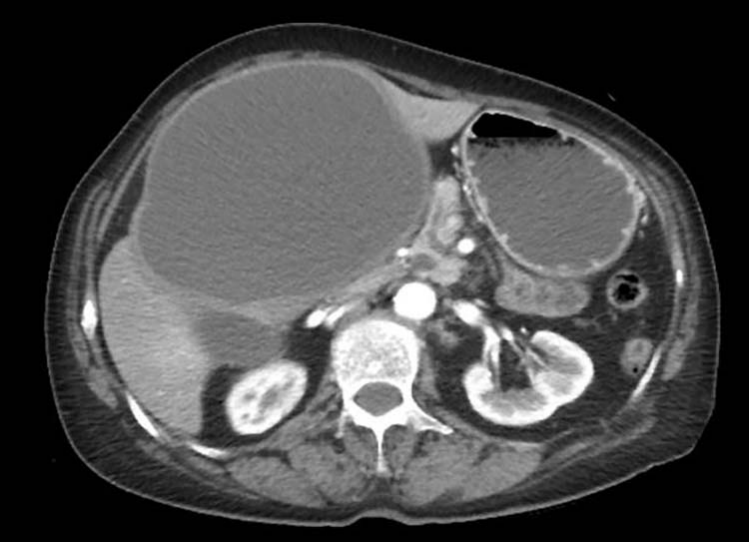
Giant hepatic cyst in the left lobe of the liver.

A 75-year-old bedridden woman presented to our hospital with chief complaints of epigastric pain, anorexia and a palpable mass in the epigastric region. She had a history of surgery for meningioma, but no history of abdominal surgery. Ultrasonography revealed a well-defined large cystic lesion with a long diameter of approximately 150 mm in the left liver lobe. Contrast-enhanced computed tomography showed a giant hepatic cyst with an irregular mass in liver segment 4, 20 × 16 cm in size (Fig. 1).

**Figure 2:**
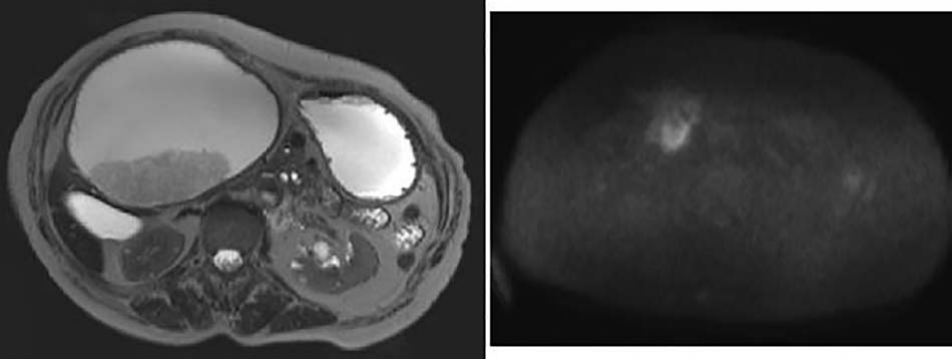
Giant cyst showed high signal intensity on T2-weighted images and fuller component in dorsal side showed high signal on diffusion-weighted images.

The hepatic cyst compressed the stomach, duodenum, pancreas and gallbladder, and the descending part of the duodenum was difficult to identify because of severe thinning caused by the compression. On magnetic resonance imaging, the giant cyst showed low signal intensity on T1-weighted and high signal intensity on T2-weighted images, and there was a solid component with a high signal on diffusion-weighted images on the dorsal side of the cyst (Fig. 2).

**Figure 3:**
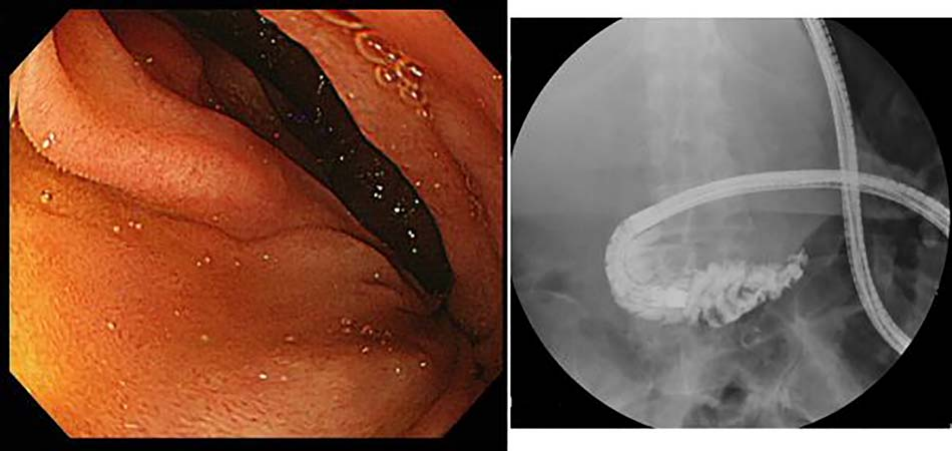
Upper gastrointestinal endoscopy showed severe stenosis of the duodenum.

Upper gastrointestinal endoscopy showed severe duodenal stenosis due to compression from outside of the wall, which was the cause of gastric pain and anorexia (Fig. 3).

The differential diagnosis included a simple liver cyst, hepatic mucinous cystic tumor and hepatic xanthogranuloma. Although we could not rule out malignancy of the intracystic tumor, we considered that hepatic resection would be too invasive for the patient. Therefore, we planned to perform laparoscopic cyst drainage to prevent dissemination and, subsequently, hepatic resection or laparoscopic deroofing depending on the puncture fluid cytology findings.

**Figure 4:**
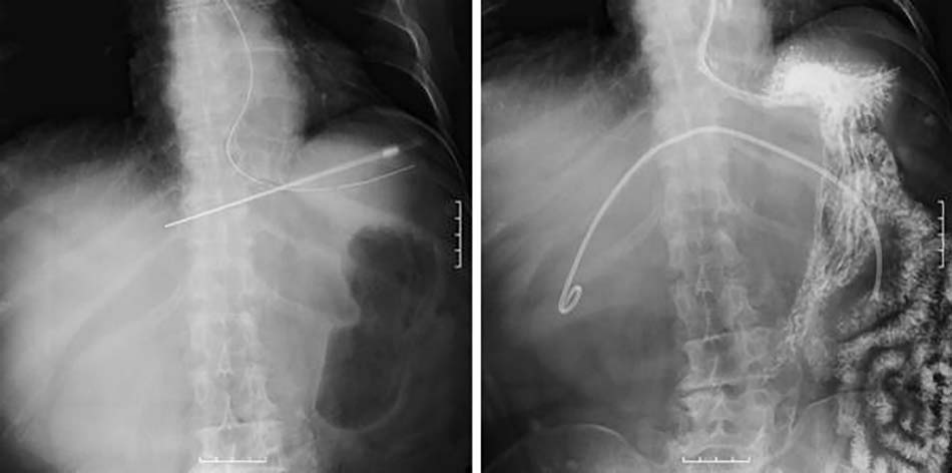
Percutaneous puncture drainage improved the obstruction of duodenum.

However, during hospitalization, the patient developed dehydration, acute renal failure, electrolyte imbalance and impaired consciousness. C-reactive protein level was elevated (9.61 mg/dL), indicating intracystic infection. As emergency treatment, percutaneous puncture drainage of the cyst was performed to decompress the stomach and duodenum (Fig. 4).

A total of 1400 mL of purulent fluid was drained, and cytology of the drainage fluid showed no malignant findings. The gastrointestinal obstruction symptoms improved, enabling oral food intake; thus, we decided to perform laparoscopic deroofing of the hepatic cyst.

**Figure 5:**
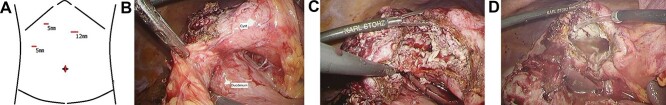
(**A**) Surgical port arrangement. (**B**) The wall of the hepatic cyst was tightly adherent to the duodenum. (**C**) The cyst wall was divided using ultrasonic coagulation incision device. (**D**) Washing inside the cyst.

**Figure 6:**
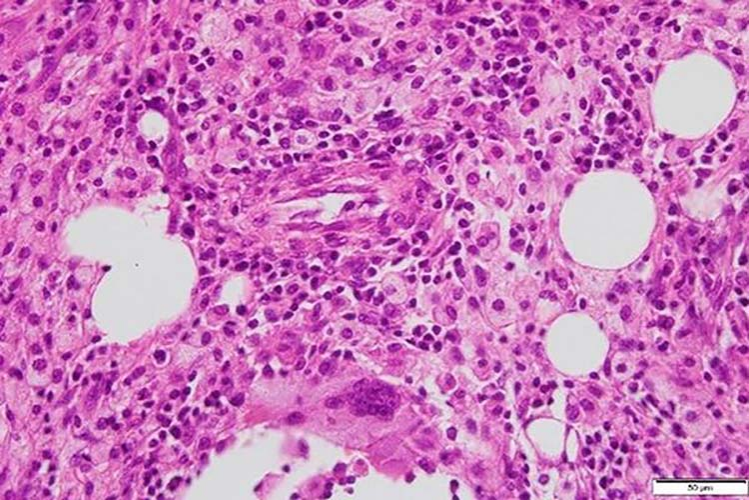
Foamy macrophages and other cells including lymphocytes and plasma cells are present.

We used three ports (one 12-mm and two 5-mm ports; Fig. 5A) for the laparoscopic approach. The cyst wall was tightly adherent to the duodenum and was difficult to dissect (Fig. 5B); therefore, it was divided without dissection using an ultrasonic coagulation incision device (Fig. 5C). We washed the inside of the hepatic cyst (Fig. 5D) and placed a drainage tube. Pathological examination of the cyst wall revealed xanthogranuloma (Fig. 6).

**Figure 7:**
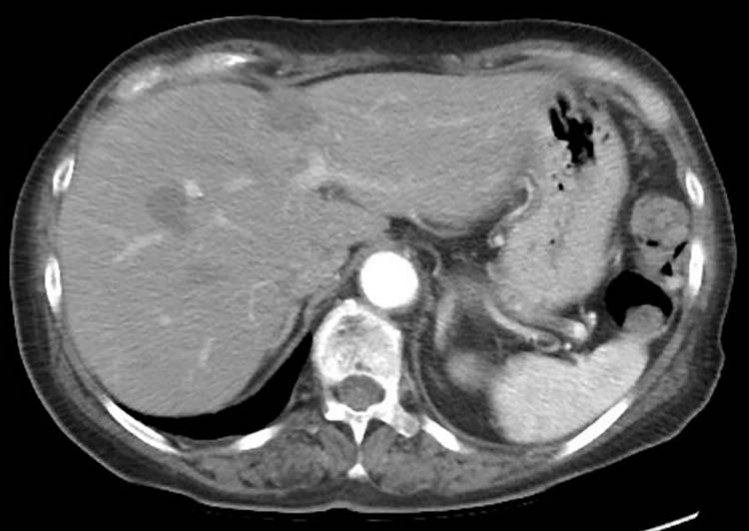
CT scan at 3 months postoperatively showed no recurrence.

The postoperative course was uneventful, and the patient was discharged on the eighth postoperative day. Follow-up computed tomography at 3 months postoperatively showed no recurrence of the liver cyst (Fig. 7).

## DISCUSSION

Cysts in the liver parenchyma border both the vascular and biliary systems and may cause inferior vena cava obstruction [5], Budd-Chiari syndrome [6], obstructive jaundice [7] and portal hypertension [8]. However, gastrointestinal obstruction is rare. Pubmed search for ‘duodenal obstruction hepatic cyst’ revealed only one similar case report [2]. In that case, puncture aspiration of the cyst and sclerotherapy with hypertonic saline were performed, but the cyst recurred 6 months later and deroofing was performed. In our case, the patient had no recurrence for 3 months after deroofing.

Large symptomatic cysts require further evaluation, as accurate diagnosis is essential for selecting appropriate treatment. Imaging is the primary modality for diagnosing simple hepatic cysts [9]. In this case, the magnetic resonance imaging findings suggested neoplastic potential, but differentiation based on preoperative imaging is difficult. In general, when a potentially malignant tumor is found in a liver cyst, the treatment options are total ablation, hepatic resection or cyst enucleation [4, 10].

In our case, the prognostic nutritional index (PNI) was under 40 (36.8), suggesting that hepatectomy would potentially be too invasive for the patient [11]. To reduce the cyst size and determine whether it is benign or malignant, cyst puncture was prioritized. Percutaneous puncture would pose a risk of seeding tumor cells; therefore, we planned to perform low-risk cytology by laparoscopic puncture and decide on a radical procedure depending on the results. However, acute renal failure due to intracystic infection forced us to perform an emergency percutaneous cyst puncture and drainage. This led to resolution of the gastrointestinal obstruction and allowed oral food intake, resulting in improved PNI of 44. Cytological examination of the puncture fluid revealed no malignant findings. Therefore, we decided to perform laparoscopic deroofing next.

In the deroofing procedure, the cyst was opened into the peritoneal cavity, and a portion of the wall was excised flush with the adjacent liver parenchyma. However, the cyst wall tightly adhered to the duodenum. As dissection of structures tightly adherent to the duodenum was a risk factor for secondary organ injury in cases of gallbladder-duodenal fistulas [12], we divided the cyst wall into two parts, leaving the part from the duodenal side. In case of a large hepatic xanthogranuloma that obstructs the duodenum and has strong adhesions, partial resection of the cyst wall may be effective.

In conclusion, a two-stage approach of cyst puncture followed by surgery may be an option for patients requiring urgent treatment for potentially malignant hepatic cysts.
